# Inhibition of Virulence Gene Expression in *Salmonella* Dublin, *Escherichia coli* F5 and *Clostridium perfringens* Associated With Neonatal Calf Diarrhea by Factors Produced by Lactic Acid Bacteria During Fermentation of Cow Milk

**DOI:** 10.3389/fmicb.2022.828013

**Published:** 2022-05-12

**Authors:** Gang Liu, Martin Laage Kragh, Søren Aabo, Annette Nygaard Jensen, John Elmerdahl Olsen

**Affiliations:** ^1^College of Veterinary Medicine, Qingdao Agricultural University, Qingdao, China; ^2^Department of Veterinary and Animal Sciences, Faculty of Health and Medical Sciences, University of Copenhagen, Frederiksberg, Denmark; ^3^National Food Institute, Technical University of Denmark, Lyngby, Denmark

**Keywords:** calf diarrhea, *Salmonella* Dublin, *Escherichia coli* F5, *Clostridium perfringens*, virulence inhibition

## Abstract

Diarrhea is a major health problem in neonatal and young calves worldwide. It can be caused by a variety of infectious agents, including the bacteria *Salmonella enterica* serovar Dublin (*S*. Dublin), enterotoxigenic *Escherichia coli* (ETEC), and *Clostridium perfringens*. Preventive alternatives to antibiotic treatment should be identified. As a first step toward this, the aim of the current study was to examine whether cell-free supernatants from cow milk fermented by lactic acid bacteria affects virulence-gene expression in strains of *S*. Dublin, ETEC *E. coli* F5 and *C. perfringens*. pH-neutralized, cell-free, spent medium of milk (nCFSM) fermented by 61 different lactic acid bacteria (LAB) and non-LAB starter cultures belonging to 17 genera was assayed for their effect on expression of important virulence factors (*S*. Dublin *hilA, ssrB, ssaG, flhD, prgI, fliC*; ETEC *E. coli* F5 *fanC, estA, fim41a*; *C. perfringens cpa*), when the bacteria were grown in the nCFSM. Screening was done using either a promoter-reporter expression system or RT-qPCR. nCFSM from *Bifidobacterium longum* BL-15955 and *Limosilactobacillus reuteri* LR-33016 downregulated the expression of *fanC, fim41a* and *estA* genes in the four tested ETEC *E. coli* F5 strains without affecting their growth, while mainly *B. longum* BL-15955 downregulated expression of *cpa* in the four tested strains of *C. perfringens*. nCFSM from the mixed cultures; NU-TRISH^®^ BY-Mild (*Lactobacillus delbrueckii* subsp. *bulgaricus, Streptococcus thermophilus* and *Bifidobacterium* BL-15954) and COMBO4 (*Lactobacillus delbrueckii* subsp. *bulgaricus* and *Streptococcus thermophilus*), as well as *Lactobacillus helveticus* CNRZ32 downregulated the tested virulence genes in the three tested strains of *S*. Dublin. To enable possible downregulation of the expression of virulence genes in all three target bacteria simultaneously, nCFSM was prepared from NU-TRISH^®^ By-Mild in combination with *B. longum* BL-15955 (i.e. a four-strain combination). The nCFSM from this combination downregulated the virulence genes expression in all the three species. In the future, NU-TRISH^®^ By-Mild and *B. longum* BL-15955 in combination could potentially be used for prevention of neonatal calf diarrhea caused by *S*. Dublin, *E. coli* F5, and *C. perfringens*, reducing the need for antimicrobial treatment, however, field studies are needed to prove that.

## Introduction

Diarrhea is common in young calves causing severe welfare problems and economic losses to cattle producers worldwide. Ten different enteric pathogens are recognized as either major [(bovine rotavirus (BRV), bovine coronavirus (BCoV), bovine viral diarrhea virus (BVDV), *Salmonella enterica, Escherichia coli, Clostridium perfringens, Cryptosporidium parvum* and *Eimeria*] or emerging pathogens [(bovine norovirus (BNoV) and bovine torovirus (BToV)] of diarrhea (Smith, 2009; Cho and Yoon, 2014). Antimicrobial treatment may lead to development of antimicrobial resistance, which threatens public health as well as future treatment possibilities against the pathogens of calf diarrhea. Alternatives to antimicrobial treatment for control of the disease are highly needed. Inhibition of the virulence factors which enables the bacteria to cause diarrhea have been suggested as a possibility (Rasko and Sperandio, 2010).

Among the bacterial pathogens, strains with particular traits dominate. In *E. coli*, strains that produce the K99 (F5) fimbriae and the heat-stable enterotoxin, STa, are traditionally associated with the disease and are known to cause more severe diarrhea than other types. Among *Salmonella* types, the cattle-adapted serovar, *S*. Dublin is the most commonly reported and economically important one (Nataro and Kaper, 1998; Acha et al., 2004; Cho and Yoon, 2014). *S*. Dublin, unlike most other *Salmonella* serovars, tends to cause systemic disease. It invades in the intestine using a Type Three Secretion system (TTSS) encoded from *Salmonella* pathogenicity island 1. From there it spreads to systemic sites, where it can survive intracellularly due to genes from another TTSS, encoded from *Salmonella* pathogenicity island 2. Pathology of the third bacterial species, *C. perfringens*, depends on toxin production. Calf diarrhea is in particular associated with strains of toxin types B and C, but type A can also be associated with the diseases in rare cases. The α toxin is the main lethal toxin shared between these three types. It promotes cell lysis through the hydrolysis of membrane phospholipids (Songer, 1997; Perez et al., 1998).

Lactic acid bacteria (LAB) are a group of Gram-positive, lactic acid producing Firmicutes. They have been used for centuries as starter or adjunct cultures in dairy fermentations. Milk proteins are a major source of bioactive peptides and an increasing number of bioactive peptides have been identified in milk protein hydrolysates and fermented dairy products (Nagpal et al., 2011). The breakdown of milk proteins by LAB plays an important role in generating peptides and amino acids for bacterial growth and in the formation of metabolites that contribute to flavor formation of fermented products. Recent studies indicate that degradation products from certain LAB, as well as some *Bifidobacterium* strains affect expression of virulence-associated genes in specific pathogens. For instance, probiotic *Lactobacillus acidophilus* La-5 and *Bifidobacterium longum* NCC2705 strains have the ability to downregulate virulence genes (*ciaB* and *flaA*) expression in *Campylobacter jejuni* (Mundi et al., 2013). Likewise, *B. bifidum* ATCC25921, *B. bifidum* BBA1, *B. crudilactis* FR/62/B/3, and *L. acidophilus* La-5 produce metabolites inhibiting virulence gene expression of enterohemorrhagic *E. coli* O157:H7 (Medellin-Pena et al., 2007; Medellin-Pena and Griffiths, 2009; Zeinhom et al., 2012) and *S*. Typhimurium (Zeinhom et al., 2012; Bondue et al., 2016). *L. acidophilus* GP1B has been shown to cause downregulation of virulence genes in *Clostridium difficile* (Yun et al., 2014), and *L. bulgaricus* NRRL B548, *Lacticaseibacillus rhamnosus* NRRL B442, *L. paracasei* DUP-13076, and *L. helveticus* LH-2 affect attachment and invasion of *Salmonella in vitro* and *in vivo* through significant inhibition of the virulence genes expression (Tellez et al., 2011; Bayoumi and Griffiths, 2012; Muyyarikkandy and Amalaradjou, 2017; Ali et al., 2019).

Based on this we hypothesized that factors produced during fermentation of cow-milk by LAB and non-LAB starter cultures could inhibit virulence factors in *S*. Dublin, ETEC *E. coli* F5 and *C. perfringens* of importance for induction of diarrhea in young calves. The aim of the study was to determine the effects of spent milk fermented by different probiotic strains on virulence gene expression in these bacteria, and to identify one or more combinations of probiotic strains, which when pooled could prevent expression of the virulence genes in all three bacteria simultaneously.

## Materials and Methods

### Bacterial Strains and Growth Conditions

The strains of pathogenic bacteria used (Table 1) were stored at −80 °C in 15% glycerol. Strains were whole genome sequenced (WGS) and analyzed for presence of virulence genes as previously described (Thomas et al., 2017). *S. Dublin* and *E. coli* F5 strains were cultivated aerobically in Luria-Bertani (LB) (Sigma, Copenhagen, Denmark) broth overnight at 37°C with shaking at 180 rpm. *C. perfringens* strains were cultivated anaerobically in Brain Heart Infusion (BHI) broth (Sigma, Copenhagen, Denmark) overnight at 37°C in an anaerobic jar plus Oxoid™ AnaeroGen™ 2.5L Sachets (ThermoFisher Scientific, Waltham, MA, USA) without shaking. A bioluminescent reporter strain of *Salmonella* Typhimurium *enterica* serovar LT2 containing a *hilA*::*luxCDABE* construct (Bayoumi and Griffiths, 2010) was grown overnight at 37 °C in LB broth supplemented with 50 μg/mL of ampicillin (Amp) (Sigma, Copenhagen, Denmark) to an approximate cell density of 1 × 10^9^ cells mL^−1^.

**Table 1:** Strains of pathogenic bacteria used in this study.

**Strain or construct**	**Serotype**	**Relevant virulence genes/genotype^**a**^**	**References**
*E. coli* E21-79	H10:O9	*fanC, fim41a, estA*	This study
*E. coli* E38-72	H37:O101	*fanC, fim41a, estA*	This study
*E. coli* E242-3	H9:O101	*fanC, fim41a, estA*	This study
*E. coli* LG3	H10:O101	*fanC, fim41a, estA*	This study
*S*. Dublin MS14334	*S*. Dublin	*hilA, prgI, ssrB, ssaG, flhD, fliC*	(Knudsen et al., 2011)
*S*. Dublin MS17265	*S*. Dublin	*hilA, prgI, ssrB, ssaG, flhD, fliC*	(Knudsen et al., 2011)
*S*. Dublin MS17266	*S*. Dublin	*hilA, prgI, ssrB, ssaG, flhD, fliC*	(Knudsen et al., 2011)
*S*. Dublin JEO3665	*S*. Dublin	*hilA, prgI, ssrB, ssaG, flhD, fliC*	(Olsen et al., 2012)
*C. perfringens* C4-5	*C. perfringens* Type A	*Cpa*	This study
*C. perfringens* C9-3	*C. perfringens* Type A	*Cpa*	This study
*C. perfringens* C16-3	*C. perfringens* Type A	*Cpa*	This study
*C. perfringens* C17-3	*C. perfringens* Type A	*Cpa*	This study
*S*. Typhimurium *hilA::lux*	LT-2	P*_*hilA*_, lux*CDABE, Amp^R^	(Bayoumi and Griffiths, 2010)

^a^*Whole genome sequences of the strains are deposited at the European Nucleotide Archive under study accession number PRJEB46410 with individual strain accession numbers*.

A total of 61 LAB and non-LAB starter cultures, including strains belonging to 17 different genera, were used in this study (Table 2). Despite including a few non-LAB starter cultures, we will use the term LAB strains for this collection throughout the study. Fifty-one of the cultures consisted of single strains, while 10 were multi-strain cultures (RD-1, NU-TRISH^®^ By-Mild, COMBO4, COMBO1, COMBO2, MIX-1, MIX-2, MIX-3, LD20 and COMBO3). The LAB strains were cultivated under anaerobic conditions overnight at appropriate temperature (30–42°C) in modified De Man, Rogosa and Sharpe medium (mMRS; 10 g peptone from casein, 8 g beef extract, 4 g yeast extract, 5 g sucrose, 1 mL tween 80, 2 g dipotassium hydrogen phosphate, 0.5 g L-cysteine HCL, 2 g diammonium hydrogen citrate, 5 g sodium acetate, 0.2 g magnesium sulfate, and 0.04 g manganese sulfate in 1 liter distilled water) (Medellin-Pena et al., 2007). The LAB strains were enumerated using MRS Agar (ThermoFisher Scientific) after 48–72 h incubation. Bifidobacterium spp. were enumerated by pour plating using MRS agar (Oxoid LTD, Hampshire, England) with 0.5 g L-cysteine (Sigma Aldrich, St. Louis, MO, USA). A schematic overview of the general experimental design is presented in Supplementary Figure S1.

**Table 2:** Lactic acid bacteria strains used in this study.

**Strains^**a**^**	**Abbr**.	**Incubation temperature (**°**C)**	**Source**
(+) *Bifidobacterium lactis, Lactobacillu delbrueckii* subsp. *bulgaricus*, *Streptococcus thermophilus*	NU-TRISH^®^ By-Mild	42	Chr. Hansen A/S
*Bifidobacterium animalis* subsp. *lactis*	BLC1	37	SACCO
*Bifidobacterium animalis* subsp. *lactis*	BL-15954	37	Chr. Hansen A/S (DSM15954)
*Bifidobacterium longum* subsp. *infantis*	BI-33361	37	Chr. Hansen A/S (DSM33361)
*Bifidobacterium longum* subsp. *longum*	BL-15955	37	Chr. Hansen A/S (DSM15955)
*Enterococcus faecium-*669	EF-669	37	Chr. Hansen A/S
*Enterococcus faecium-*202	EF-202	37	Chr. Hansen A/S
*Enterococcus faecium-*339	EF-339	37	Chr. Hansen A/S
*Kocuria varians*	KV	37	This study
(+) *Kocuria varians, Latilactobacillus cuvatus, Staphylococcus carnosus*	RD-1	37	Frutarom
*Lactobacillus acidophilus*	LA-3	37	SACCO
*Lactobacillus acidophilus*	LA-13241	37	Chr. Hansen A/S (DSM13241)
*Lactobacillus acidophilus*	LA-20079	37	DSM20079
*Ligilactobacillus animalis-*506	LA-506	37	Chr. Hansen A/S
*Lentilactobacillus buchneri-*881	LB-881	37	Chr. Hansen A/S
*Lactobacillus delbrueckii* subsp. *bulgaricus*	SP5	42	SACCO
(+) *Lactobacillus delbrueckii* subsp. *bulgaricus, Streptococcus thermophilus*	COMBO4	37	Chr. Hansen A/S
*Levilactobacillus brevis*	LB	30	Chr. Hansen A/S
*Lacticaseibacillus paracasei* subsp. *paracasei*	LP-33451	37	Chr. Hansen A/S (DSM33451)
*Lacticaseibacillus casei*	BGP93	37	SACCO
*Latilactobacillus curvatus*	LC	37	This study
*Lactobacillus delbrueckii*	LB-20074	37	DSM20074
(+) *Lactobacillus delbrueckii* subsp. *bulgaricus*, *Streptococcus thermophilus*	COMBO1	42	Chr. Hansen A/S
(+) *Lactobacillus delbrueckii* subsp. *bulgaricus*, *Streptococcus thermophiles*	COMBO2	37	Chr. Hansen A/S
*Limosilactobacillus fermentum*	LF	37	Chr. Hansen A/S
*Lactobacillus helveticus*	LH521	42	ATTC-521
*Lactobacillus helveticus*	CNRZ32	42	(Jensen et al., 2009)
*Lactobacillus helveticus-*02	LH-02	42	Chr. Hansen A/S
*Lactobacillus johnsonii*	LJ-10533	37	DSM10533
*Lacticaseibacillus paracasei*	BGP1	37	SACCO
*Lacticaseibacillus paracasei*	BGP2	37	SACCO
*Lacticaseibacillus paracasei*	LMG P-17806	37	Chr. Hansen A/S
*Lacticaseibacillus paracasei*	LP-20006	37	DSM20006
*Lactiplantibacillus plantarum*	LPAL	37	SACCO
*Lactiplantibacillus plantarum-*672	LP-672	37	Chr. Hansen A/S
*Lactiplantibacillus plantarum-*673	LP-673	37	Chr. Hansen A/S
*Lactiplantibacillus plantarum-*072	LP-072	37	Chr. Hansen A/S
*Lactiplantibacillus plantarum*	LP-20174	30	DSM20174
*Limosilactobacillus reuteri*	LR-33016	37	Chr. Hansen A/S (DSM33016)
*Limosilactobacillus reuteri*	LR-20016	30	DSM 20016
*Lacticaseibacillus rhamnosus*	IMC 501	37	SACCO
*Lacticaseibacillus rhamnosus*	LR-33156	37	Chr. Hansen A/S (DMS33156)
*Lacticaseibacillus rhamnosus*	LR-20021	37	DSM20021
*Lacticaseibacillus rhamnosus*	SP1	37	SACCO
*Lactococcus lactis*-955	LL-995	30	Chr. Hansen A/S
*Lactococcus lactis*-671	LL-671	30	Chr. Hansen A/S
(+) *Lactococcus cremoris,Leuconostoc, Lactococcus lactis* subsp. *lactis, Lactococcus lactis* subsp. *Lactis biovar diacetylactis*	MIX-1	30	Chr. Hansen A/S
(+) *Lactococcus cremoris, Leuconostoc, Lactococcus lactis* subsp. *lactis, Lactococcus lactis* subsp. *Lactis biovar diacetylactis*	MIX-2	30	Chr. Hansen A/S
(+) *Lactococcus cremoris, Lactococcus lactis* subsp. *lactis, Lactococcus lactis* subsp. *Lactis biovar diacetylactis, Leuconostoc* spp.	MIX-3	30	Chr. Hansen A/S
*Lactococcus lactis* spp. *lactis* biovar diacetylactis (*L. diacetylactic*)	LD	37	Chr. Hansen A/S
(+) *Latilactobacillus sakei* subsp. *sakei, Staphylococcus carnosus*	LD-20	37	BiTEC
(+) *Lactobacillus delbrueckii* subsp. *bulgaricus, Streptococcus thermophiles*	COMBO3	37	Chr. Hansen A/S
*Pediococcus acidilactici*-839	PA-839	37	Chr. Hansen A/S
*Pediococcus pentosaceus*-354	PP-354	30	Chr. Hansen A/S
*Pediococcus pentosaceus*-670	PP-670	30	Chr. Hansen A/S
*Pediococcus pentosaceus*-674	PP-674	30	Chr. Hansen A/S
*Propionibacterium freudenreichii* subsp. *shermanii*	PF7	30	SACCO
*Propionibacterium freudenreichii* subsp. *shermanii*	PF8	30	SACCO
*Propionibacterium freudenreichii* subsp. *shermanii*	PB-1	30	SACCO
*Propionobacterium freudenreichii-*507	PF-507	30	Chr. Hansen A/S
*Staphylococcus carnosus*	SC	37	This study

^a^*Multi-strain cultures indicated with (+)*.

### Fermentation of Cow Milk

Neutralized-cell-free spent medium (nCFSM) was prepared as previously described (Medellin-Pena et al., 2007) with minor modifications. Briefly, the LAB strains were seeded from agar media and grown anaerobically in milk for 16 h at appropriate temperature and then subcultured in sterilized 3.5% whole milk (Thise Dairy, Denmark) using 10% inoculum and 18 h fermentation according to the results of preliminary tests described below. The sterilization of milk was carried out by heating in a water bath at 95°C for 30 min. The fermented milk samples were first centrifuged at 18,500 × g for 3 min at 20°C, and the coagulated fat layer at the top of the tube was removed with a sterile spoon. Then the cells were precipitated by centrifugation at the same conditions for 10 min at 20 °C, the supernatants were harvested and neutralized to pH 7.2 ± 0.2 with 6 M NaOH in order to avoid effects caused merely by acidification. The pH adjusted supernatants were further centrifuged for 5 min to remove all coagulated fat layer to enable easy filter-sterilization through a 0.2-um-pore-size filter (Millipore) by vacuum to remove live bacteria. As a control, nCFSM from unfermented milk was prepared as described above including pH adjustment to 7.2. nCFSM preparations were stored at−20 °C prior to use.

The potential of LAB fermented milk to elicit inhibition of virulence factors in pathogenic bacteria may depend on the fermentation conditions. Therefore, optimal fermentation time and percent inoculation for this was determined in preliminary experiments using whole milk 3.5% fat (Thise Dairy, Denmark) or reconstituted milk powder (Thise Dairy, Denmark). The milk/reconstituted milk powder were fermented with four different fermentation protocols with 1% and 10% v/v inoculation level of the starter culture combined with either 6 h or 18 h fermentation time using three different starter cultures (*L. helveticus* CNRZ32, *L. acidophilus* LA-13241 and *L. rhamnosus* LR-33156).

In order to understand the relation between growth of LAB and the final pH in the fermented milk, 10 selected LABs were seeded to pasteurized whole milk at 10^6^-10^7^ CFU/mL and incubated at the optimal growth temperature for 18 hours. At this time, CFU and pH was determined as described above. Determinations were done in triplicate. The strains were selected to include the strains used in the experiment described above (CNRZ32, LA-13241 and LR-33156), five of the 10 strains used later in the more detailed characterization of the inhibitory effect on virulence gene expression (BL-15955, NU-TRISH^®^ By-Mild, COMBO4, CNRZ32, LR-33016) and three additional strains (BL-15954, LP-673 and LMG P-17806).

### Effect of NCFSM on *HilA* Gene Expression in *S*. Typhimurium LT2 Reporter Construct

The putative anti-virulence effects was first assayed using the bioluminescent reporter strain *S*. Typhimurium LT2 *hilA::luxCDABE* (Bayoumi and Griffiths, 2010), where the expression of the *lux* genes is controlled by the *hilA* promoter, so that light emits when the *hilA* gene is expressed. *S*. Typhimurium LT2 *hilA::luxCDABE* was grown in nCFSM, and expression of *hilA* was measured as previously reported (Muyyarikkandy and Amalaradjou, 2017; Ali et al., 2019). Briefly, an overnight culture (19 h) of the reporter strain with an approximate cell density of 1 × 10^9^ cells mL^−1^ was diluted 1:100 in nCFSM supplemented with 10% (v/v) 10x LB medium. Then 200 μl of each inoculated nCFSM was distributed into triplicate wells of a sterile opaque corning 96-well plate (Sigma-Aldrich), which was placed in a Victor multi-lable counter (Wallac, PerkinElmer Life Sciences Canada, Woodbridge, ON, Canada) for incubation at 37 °C. The growth of the reporter strain expressed by cell density (OD_490_) and *hilA* gene expression measured as bioluminescence were measured hourly for 24 h. The bioluminescence was expressed as relative light units (RLU) defined as bioluminescence counts min^−1^ and adjusted by OD_490_ (RLU/OD_490_). Accumulated gene expression over 12 h was calculated by summarizing bioluminescence signal for the first 12 h of measuring and termed Sum (12 h). The proportional change in expression of *hilA* in *S*. Typhimurium LT2 *hilA::luxCDABE* construct has been termed change in virulence activity (%VA) and was calculated as 100 – [(expression in nCFSM / expression in the non-fermented milk (control)] ^*^ 100. Since this assay was only used for initial screening and results were confirmed by RT-PCR, only one biological assay with two technical replicates was performed.

### QPCR Measurement of the Effect of NCFSM on Transcription of Virulence Genes in *S*. Dublin, *E. Coli* F5 and *C. Perfringens*

#### Culture of Pathogens in NCFSM

Single colonies of *E. coli* and *S. Dublin* were grown in LB broth aerobically with shaking (180 rpm), and *C. perfringens* were grown in BHI broth anaerobically in anaerobic jars that utilized Oxoid™ AnaeroGen™ 2.5 L Sachets (ThermoFisher Scientific, Waltham, MA, USA) without shaking overnight at 37°C. The overnight cultures were diluted to a final OD_600_ = 0.05 by mixing with 4.5 mL nCFSM supplemented with 0.5 mL of 10x appropriate broth (LB or BHI) and then grown at 37°C to early stationary phase (OD_600_ =1.0) as recommended (Bayoumi and Griffiths, 2012). To serve as controls, the overnight cultures were similarly diluted into unfermented milk supplemented with LB or BHI and grown to OD_600_ =1.0.

#### RNA Extraction

RNA was isolated using a FastPrep cell disrupter system (Qbiogene, Illkirch, France) and RNeasy Mini Kit (Qiagen, Sollentuna, Sweden) for total RNA isolation by mechanical disruption, according to the manufacturer's instructions. Quantity of the extracted RNA was determined by A260 measurements and purity by A260/A280 ratio measurements using a NanoDrop 1000 spectrophotometer (Thermo Scientific, Hvidovre, Denmark). RNA samples were purified by DNA digestion using TURBO^TM^ DNase kit (2 U/μl) (Ambion, Life Technologies, Nærum, Denmark) to remove contaminating DNA.

#### Reverse-Transcribed-Quantitative Real-Time Polymerase Chain Reaction (RT-QPCR)

RNA was reverse-transcribed into cDNA using the High Capacity cDNA Reverse Transcription Kit (Life Technologies, Nærum, Denmark) under the following conditions: 25°C for 10 min, 37°C for 120 min, 85 °C for 5 min, and a cooling step to 4°C. RT-qPCR was performed using FastStart Essential DNA Green Master (Roche, Hvidovre, Denmark). For *E. coli* and *C. perfringens* RT-qPCR were performed using a LightCycler 96 (Roche, Hvidovre, Denmark), where as an Applied Biosystems QuantStudio 5 (Thermo Fisher Scientific, Roskilde, Denmark) were used for *S. Dublin*. The standard qPCR cycling program was as following: initial activation denaturation (95 °C for 10 min), and followed by 40 cycles (95°C for 15 s, 60°C for 30 s, 72°C for 30 s).

Expression levels of the virulence factors listed in Table 1 were measured. Primers are listed in Supplementary Table S1. Real-time PCR amplification efficiencies were calculated from the slope of the standard curve, which was constructed using the data collected from serial dilutions of the template DNA for each gene according to the equation: *E* = 10 ^[−1/slope]^ (Pfaffl, 2001). The transcript levels were normalized to validated reference genes, *gapA, nusG, 16S rRNA* and *GAPDH* (Wise and Siragusa, 2005; Moller et al., 2017; Shi et al., 2019). RT-qPCR was performed on three biological replicates with two technical replicates each, and the relative changes in gene expression were calculated by using the formula: ΔCT = CT (target) – CT (normalizer), and ΔΔCT was calculated by subtracting the ΔCT of the untreated sample (pathogens grown in non-fermented milk) from the treated one (pathogen grown in nCFSM): ΔΔCT = ΔCT (treated) - ΔCT (untreated). The relative gene expression was calculated as 2^−ΔΔCT^. Finally, a fold change was calculated as −1/2^−ΔΔCT^ (Pfaffl, 2004).

### Statistical Analysis

Statistical analysis was performed using GraphPad Prism (GraphPad Software) version 7.03. Comparison of gene expression was analyzed using Student's *t*-test with Welch's correction. *P* ≤ 0.05 were considered statistically significant.

## Results

### Optimal Fermentation Protocol for Inhibition of Virulence Genes

In order to choose optimal fermentation conditions for maximizing the effect on virulence gene expression, cell free spent medium (CFSM) was prepared from whole milk and reconstituted milk powder fermented with the strains *L. helveticus* CNRZ32, *L. acidophilus* LA-13241 or *L. rhamnosus* LR-33156 based on four different fermentation protocols with variation in seeding quantity and fermentation time. After pH neutralization, the nCFSM obtained from fermented reconstituted milk powder resulted in less inhibition of the *hilA* gene expression in *S*. Typhimurium compared with nCFSM from whole milk-based fermentation (data not shown). Thus, whole milk was used in the following experiments.

The pH in CFSM from *L. helveticus* CNRZ32 was 4.5 (1% inoculation) and 4.0 (10% inoculation) after 6 h fermentation, which decreased to 3.5 (1% inoculation) and 3.0 (10% inoculation) after 18 h fermentation, respectively. CFSM from *L. acidophilus* LA-13241 showed a pH of 6.5 (1% inoculation) and 5.0 (10% inoculation) after 6 h fermentation while 18 h of fermentation decreased pH to 5.0 (1% inoculation) and 4.5 (10% inoculation). CFSM of *L. rhamnosus* LR-33156 was 6.5 (1% inoculation) and 6.3 (10% inoculation) after 6 h and was 6.0 at both inoculation levels after 18 h. For these three strains, and seven other strains tested for comparison, the final pH generally correlated inversely with the Log CFU/ml at the end of the 18 hours of fermentation (R = −0.69). In this testing the end cell density of *L. helveticus* CNRZ32 (9.1±0.1 CFU/mL) exceeded that of the other strains, and the resulting pH was below 4, while *L. rhamnosus* LR33156 had a more than one log lower end CFU/ml and a higher end pH close to 6.0 (Supplementary Figure S2). However, there were exceptions to the general correlation, for example both *B. lactis* BL-15954 and *B. longum* BL15955 has a final pH >5 with approximate 8 Log CFU/ml, while *L. plantarum* LP-673 had a pH ≈5, with a final cell concentrations at 7 Log CFU/mL, as seen in Supplementary Figure S2. To rule out that the effect of different pH could influence down-stream results, all CFSM were neutralized (nCFSM) before they were used as growth medium for pathogenic bacteria.

In general, nCFSM from *L. helveticus* CNRZ32 showed the highest inhibition of *hilA* expression in *S*. Typhimurium irrespective of fermentation protocols. The trend was that 10% inoculation level resulted in a higher inhibition of *hilA* expression than 1% inoculum, and that 18 h fermentation was superior to 6 h fermentation, however this was not uniform among the strains (Table 3).

**Table 3:** The proportional change (VA%) in expression of the virulence gene *hilA* in *S*. Typhimurium after growth in nCFSM from *L. helveticus* CNRZ32, *L. acidophilus* LA-13241, and *L. rhamnosus* LR-33156.

	**6 h fermentation (VA%)**				**18 h fermentation (VA%)**			
**LAB strains**	**1%** ^**a**^		**10%** ^**a**^		**1%** ^**a**^		**10%** ^**a**^	
	**12 h^**b**^**	**Sum (12 h)^**b**^**	**12 h^**b**^**	**Sum (12 h)^**b**^**	**12 h^**b**^**	**Sum (12 h)^**b**^**	**12 h^**b**^**	**Sum (12 h)^**b**^**
*L. helveticus* CNRZ32	−45 ± 2	−40 ± 1	−69 ± 7	−65 ± 4	−30 ± 11	−15 ± 11	−96 ± 1	−57 ± 15
*L. acidophilus* LA-13241	−3 ± 10	13 ± 8	−27 ± 1	−7 ± 2	−41 ± 14	−1 ± 5	−55 ± 4	−20 ± 3
*L. rhamnosus* LR-33156	−26 ± 10	−12 ± 10	−19 ± 11	−17 ± 13	−41 ± 15	−1 ± 10	−18 ± 11	−4 ± 13

^a^*Inoculation level*.

^b^*Inhibition of hilA expression in the hilA::luxCDABE bioluminescence reporter estimated as nCFSM relative to the non-fermented milk control at time point 12 h and as the area under the accumulated expression curve during 12 h [sum(12h)]*.

**Figure 1:**
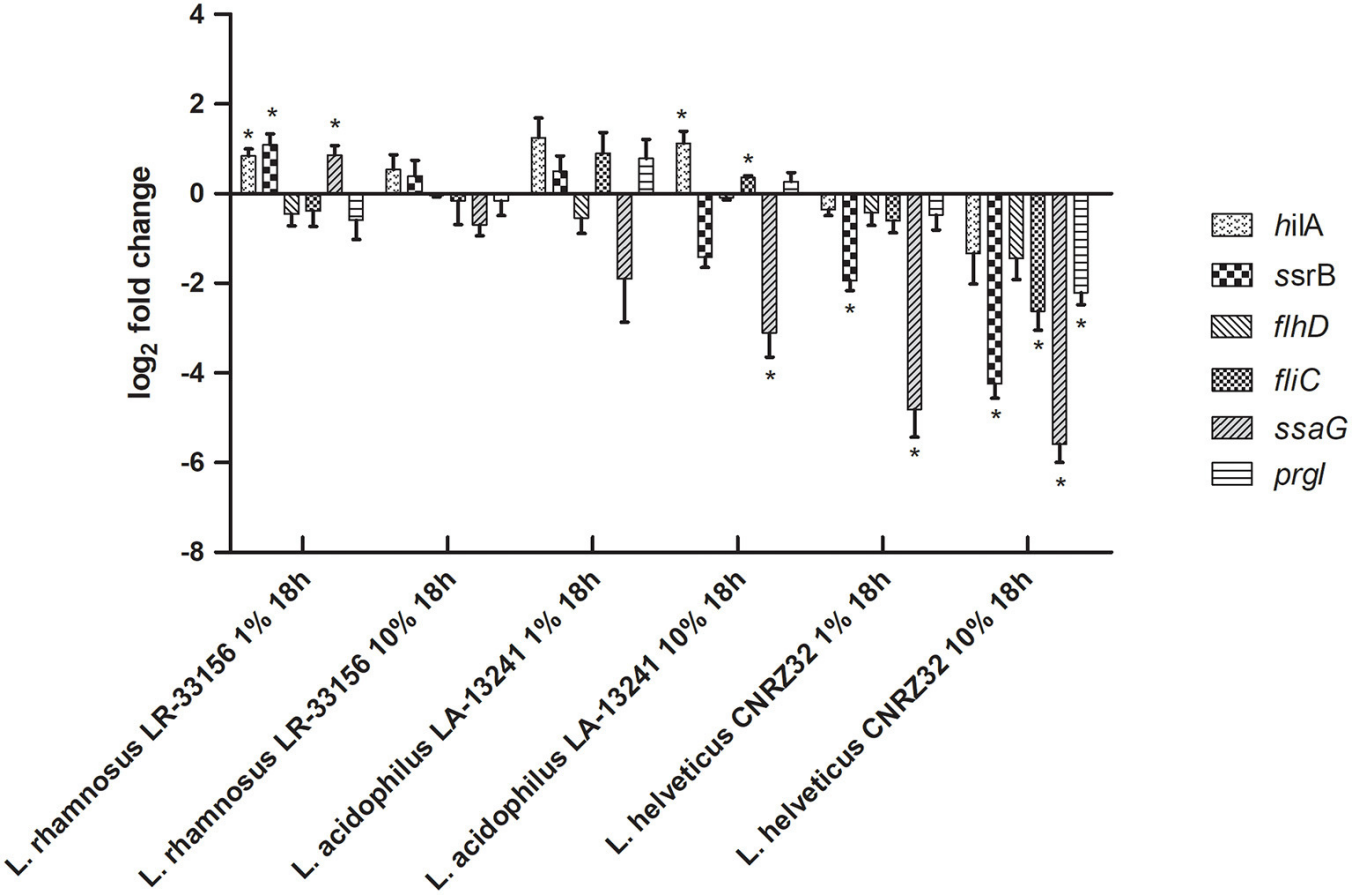
Effect of nCFSM of *L. helveticus* CNRZ32, *L. acidophilus* LA-13241 and *L. rhamnosus* LR-33156 on the expression of *hilA, ssrB, flhD, fliC, ssaG* and *prgI* in *S*. Dublin analyzed by RT-qPCR. The CFSM were prepared from milk fermented 18 h with an inoculation level of 1% and 10%. The change in expression of genes is relative to the non-fermented milk control. Three independent replicates including two technical replicates each were performed. The data shown represents the mean and the error bars represent standard deviations. The stars indicate statistical significance at different levels: ^*^P ≤ 0.05.

*S*. Typhimurium may cause calf diarrhea, but it is not the most common and most important serovar of *Salmonella* in relation to this disease, which is *S*. Dublin. Therefore, although the *S*. Typhimurium bioluminescence assay provides a relatively easy screening approach, RT-qPCR was applied to assess expression of six relevant virulence genes in *S. Dublin* after growth in nCFSM from each of the three LAB for 18 h with 10% inoculation vs. 1% inoculation. This showed variation with both up- and downregulations of virulence genes with the exception of *L. helveticus* CNRZ32, which showed consistent downregulation for all tested virulence genes of *S. Dublin* (Figure 1). *L. helveticus* CNRZ32 showed significant downregulation of *ssrB* (18.9-fold), *flhD* (2.73-fold), *fliC* (6.14-fold), *ssaG* (48.0-fold) and *prgI* (4.63-fold) at 10% inoculation level, while expression of *hilA* (2.52-fold) and *flhD* (2.73-fold) was lowered, but not significantly different from the control. Based on the results on *hilA* expression in the *S*. Typhimurium reporter strain and virulence genes in *S*. Dublin, a fermentation protocol with a 10% inoculum level of the starter cultures and a fermentation time of 18 h was applied for the onward testing of effects of nCFSMs.

### Effect of NCFSM on *HilA* Gene Expression in *S*. Typhimurium LT2 Reporter Construct

The bioluminescent reporter strain, *S*. Typhimurium LT2 *hilA*::*luxCDABE*, was used for initial screening of the effects of nCFSM obtained from milk fermented with 61 different LAB strains/combinations of LAB strains. No effects were observed on growth phenotypes of *S*. Typhimurium LT2 *hilA*::*luxCDABE* during 24 h incubation at 37°C in presence of 10% nCFSM from 59 LAB, while nCFSM from *L. paracasei* BGP1, BGP2 and *P. pentosaceus* PP-670 did not sustain good growth (OD_490_ <0.5 after 24 h).

**Figure 2:**
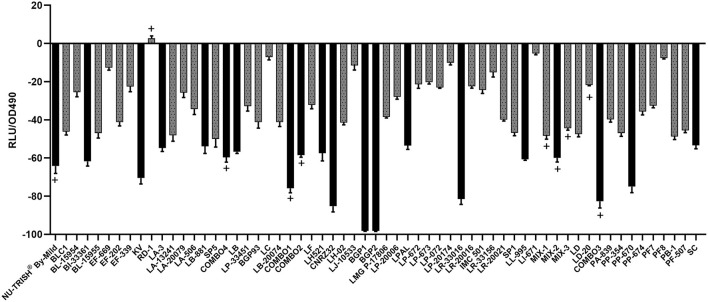
Change in bioluminescence (%VA) of *S*. Typhimurium LT2 *hilA*::*luxCDABE* after 12 h incubation in nCFSM from 61 different LAB strain cultures. Strains where growth in the nCFSM resulted in more than 50 % reduction in bioluminescence compared to growth in unfermented milk are shown as black bars. The *hilA* measurement was based on one biological replicate of nCFSM with technical triplicates. The multi-strain starter cultures are marked with (+).

Bioluminescence of the *S*. Typhimurium LT2 *hilA*::*luxCDABE* after 12 h of growth in nCFSM compared to growth in non-fermented milk (control) was reduced for all tested LAB strains except RD1 (Figure 2). nCFSM from *L. paracasei* BGP1 and BGP2 elicited the highest downregulation of *hilA* expression by 98.2%, however, due to the effect on growth phenotype, these results were dis-regarded. Other single strain starter cultures eliciting more than 50% downregulation of *hilA* expression were *L. helveticus* CNRZ32 (85.3%), *L. reuteri* LR-33016 (81.5%), *P. pentosaceus* PP-670 (74.7%), *K. varians* (70.5%), *B. infantis* BI-33361 (61.8%), *L. lactis* LL-995 (60.7%), *L. helveticus* LH521 (57.5%), *L. brevis* (57%), *L. acidophilus* LA-3 (54.8%), *L. buchneri* LB-881 (53.9%), *L. plantarum* LPAL (53.3%), and *S. carnosus* (53%) (Figure 2). For seven out of ten multi-strain starter cultures, downregulation of *hilA* expression was more than 50% [(COMBO3 (82.7%), COMBO1 (75.9%), NU-TRISH^®^ By-Mild (64.2%), MIX-2 (60.0%), COMBO4 (59.7%), COMBO2 (58.5%)] (Figure 2).

### Effect of NCFSM on Transcription of Virulence Genes in *S*. Dublin, *E. Coli* F5 and *C. Perfringens*

Even if bioluminescent reporter genes are considered a valuable tool for a first screening of gene expression, bioluminescence may be influenced by several factors, including components of the medium (Ali et al., 2019). Therefore, RT-qPCR as a highly sensitive and more specific method is necessary to complement the bioluminescence results. A two-step procedure was used. First, the effects of nCFSM from the 61 LAB strains on virulence gene expression was measured in one strain of each of *E. coli* F5 (E21-79), *S. Dublin* (JEO3665) and *C. perfringens* (C4-5) after incubation to OD_600_ = 1.0. Based on the combined results of bioluminescent and RT-qPCR analysis (Supplementary Table S2), the 10 most promising nCFSMs were selected to make sure all pathogens were covered sufficiently. Among the 10 selected nCFSMs, NU-TRISH^®^ By-Mild, *L. casei* BGP93, *L. reuteri* LR-33016, *P. acidilactici* PA-839, *P. pentosaceus* PP-674, *Pr. freudenreichii* PF-507 and *L. helveticus* CNRZ32 inhibited all tested virulence genes in *S*. Dublin and *E. coli*. *B. longum* BL-15955 and *Lentilactobacillus buchneri* LB-881 inhibited all tested virulence genes in *E. coli* and *C. perfringens*, while COMBO4 inhibited all tested virulence genes in *S*. Dublin and *C. perfringens*.

**Figure 3:**
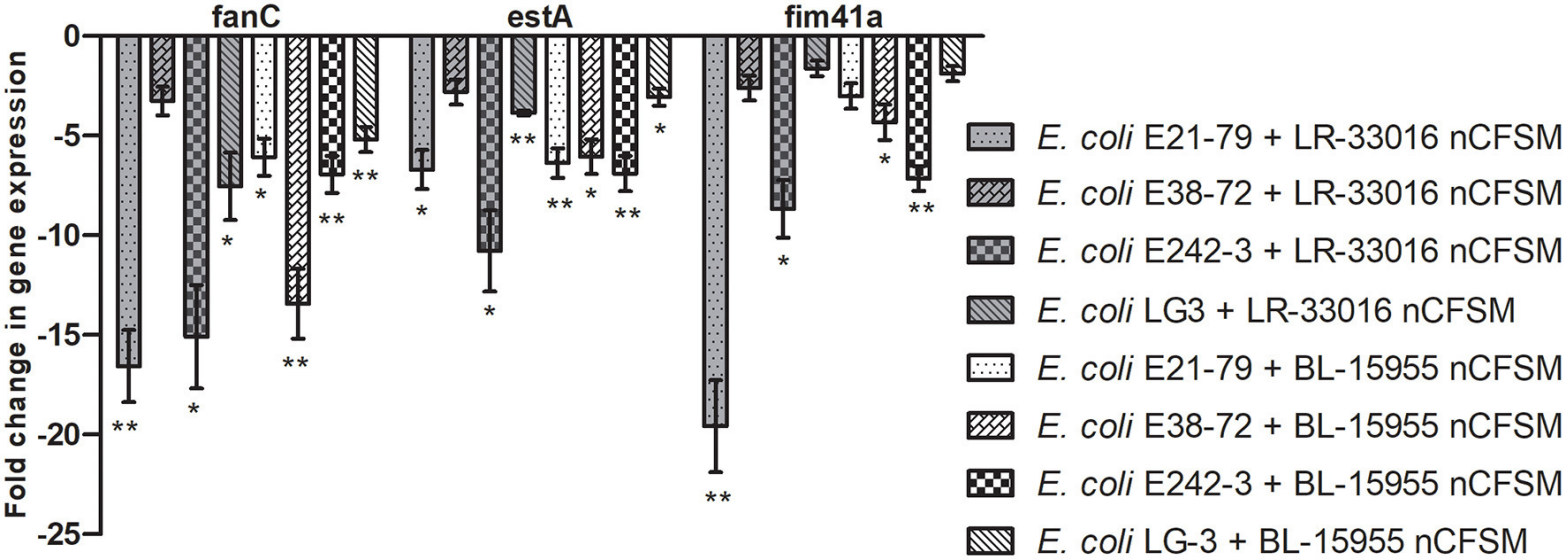
Effect of *L. reuteri* LR-33016 and *B. longum* BL-15955 nCFSM on virulence gene expression of 4 *E. coli* F5 strains presented as fold change of expression levels in nCFSM relative to non-fermented milk. The expression data were normalized to two validated reference genes, *gapA* and *nusG*. Three independent replicates including two technical replicates each were performed. The data shown represents the mean and the error bars represent standard deviations. The stars indicate statistical significance at different levels: *P ≤ 0.05, ** *P* ≤ 0.01.

**Figure 4:**
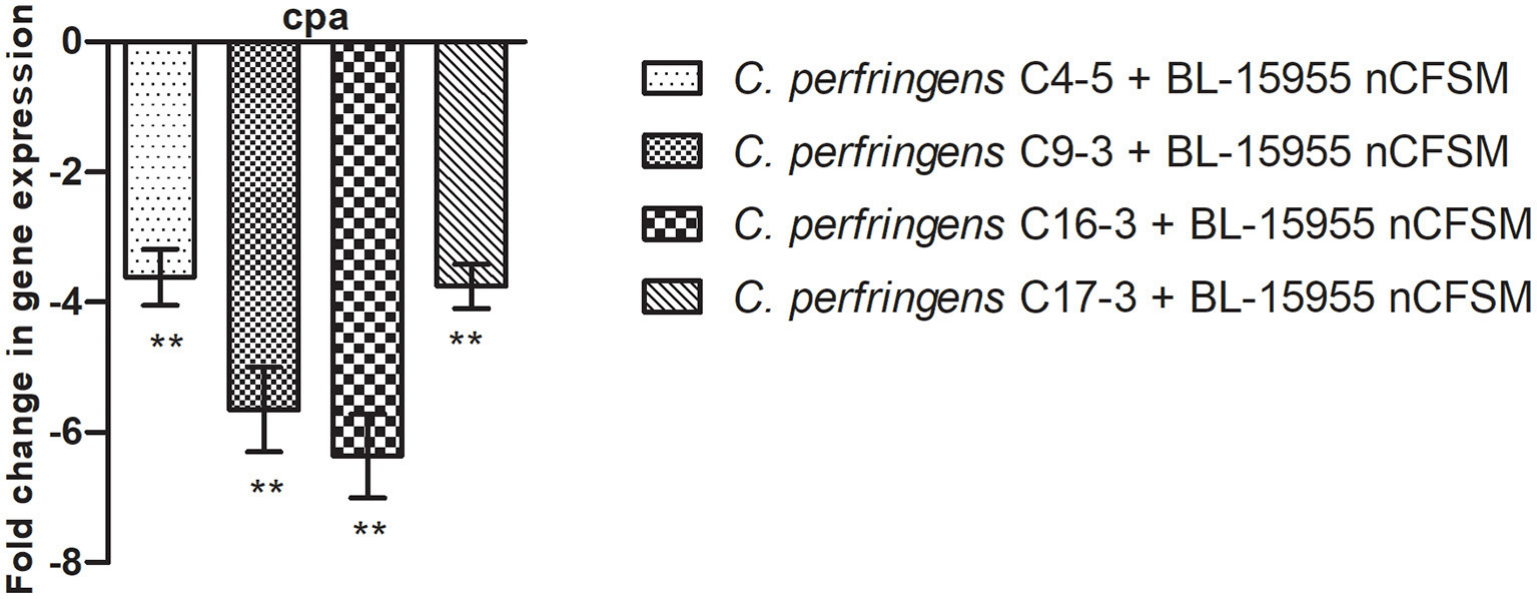
Effect of *B. longum* BL-15955 nCFSM on expression of *cpa* in 4 *C. perfringens* strains presented as fold change of expression levels in nCFSM relative to non-fermented milk. The expression data were normalized to two validated reference genes, *16sRNA* and *GAPDH*. Three independent replicates including two technical replicates each were performed. The data shown represents the mean and the error bars represent standard deviations. The stars indicate statistical significance at different levels: ** *P* ≤ 0.01.

**Figure 5:**
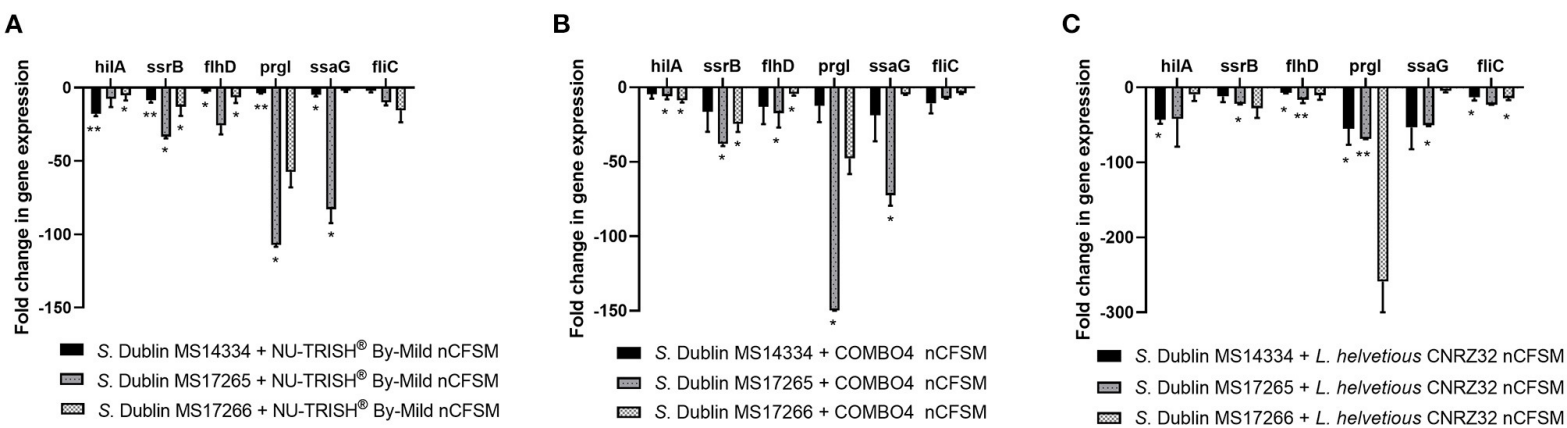
Effect of nCFSM from **(A)** NU-TRISH^®^ By-Mild (*L. delbrueckii* subsp. *bulgaricus, S. thermophilus* and *B. lactis* BL-15954, **(B)** COMBO4 (*L. delbrueckii* subsp*.bulgaricus* and *S. thermophilus*) and **(C)** *L. helveticus* CNRZ32 on virulence gene expression in 3 *S. Dublin* strains. The expression data were normalized to validated reference gene *gapA* and presented as fold change of expression levels in nCFSM relative to a control (non-fermented milk). Three independent replicates including two technical replicates each were performed. The data shown represents the mean and the error bars represent standard deviations. The stars indicate statistical significance at different levels: *P ≤ 0.05, ** *P* ≤ 0.01.

As a second step, four strains of *E. coli* F5, 3 strains of *S*. Dublin and 4 strains of *C. perfringens* were then grown in the 10 selected nCFSMs, and virulence gene expression was tested on by RT-qPCR to verify pathogen strain coverage. It was found that *fanC, fim41a* and *estA* genes were significantly downregulated in the four *E. coli* strains after growth in nCFSM from *L. reuteri* LR-33016 and *B. longum* BL-15955 (Figure 3). nCFSM from *B. longum* BL-15955 significantly downregulated *fanC* expression with 5.19 to 13.43 fold, *estA* expression with 3.06 to 6.90 fold, and *fim41a* with 1.88 to 7.15 fold. *L. reuteri* LR-33016 significantly downregulated *fanC* expression with 7.53 to 15.09 fold, *estA* expression with 3.86 to 10.78 fold, and *fim41a* with 2.62 to 19.58 fold, except in *E. coli* E38-72, where it caused a non-significant 2.62 to 3.26 fold decrease in expression (Figure 3). *B. longum* BL-15955 also significantly downregulated *cpa* expression in all four tested *C. perfringens* strains with 3.65 to 6.24 fold (Figure 4), while little effect of other nCFSMs was observed (Supplementary Dataset S1). In the 3 tested *S*. Dublin strains, two multi-strain cultures, NU-TRISH^®^ By-Mild (*L. delbrueckii* subsp. *bulgaricus, S. thermophilus* and *B. lactis* BL-15954) and COMBO4 (*L. delbrueckii* subsp. *bulgaricus* and *S. thermophilus*), as well as *L. helveticus* CNRZ32 significantly downregulated *hilA* expression with 4.6 to 43 fold, *ssrB* expression with 8.7 to 38.1 fold, *flhD* with 2.7 to 25.7 fold, *prgL* with 3.7 to 258 fold, *ssaG* with 2.0 to 82.8 fold, and *fliC* with 2.2 to 22.8 fold expression (Figure 5).

### Activity of NCFSM Cocktails on Expression of Virulence Genes in Bacterial Pathogens of Calf Diarrhea

**Figure 6:**
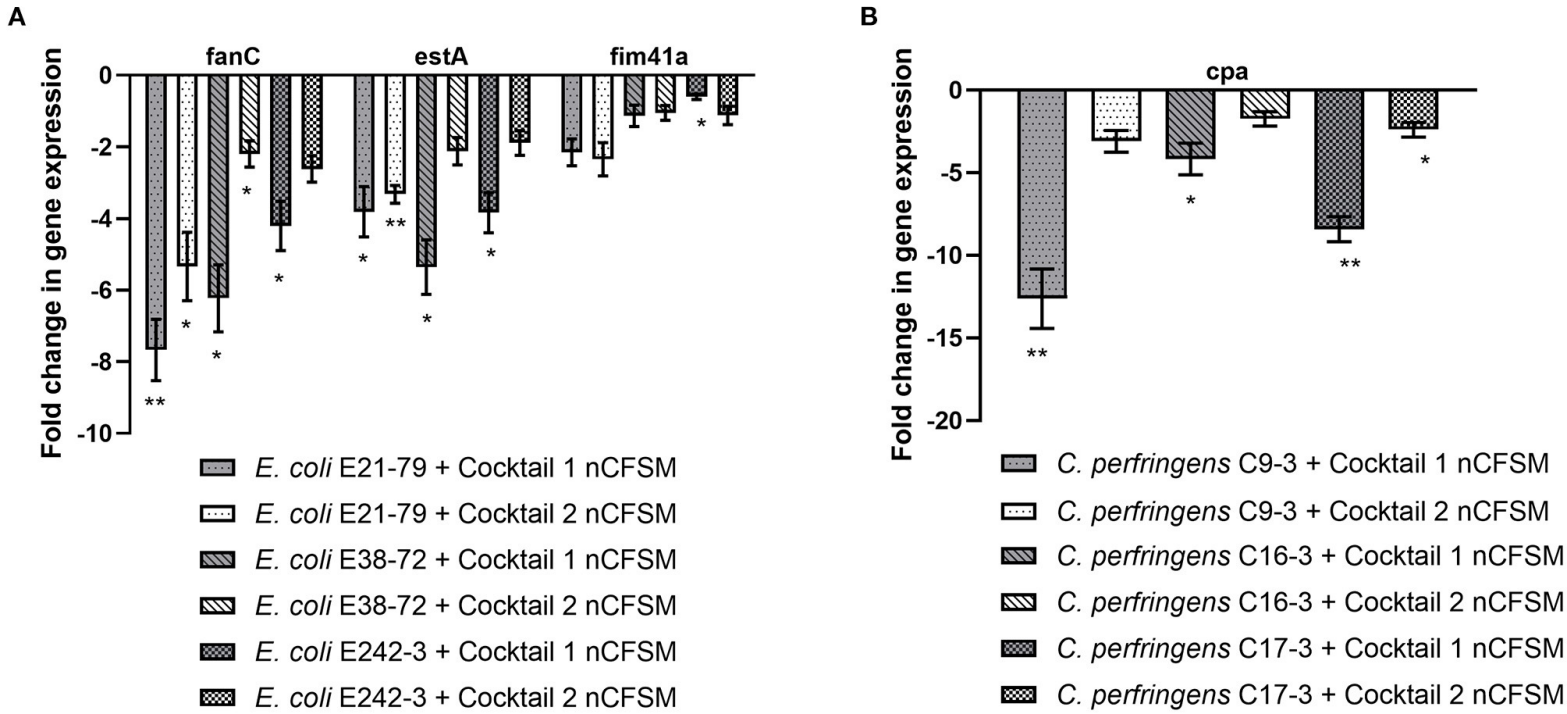
Effect of nCFSM from two cocktails of LAB strains on expression of *fanC, estA* and *fim41a* in 3 *E. coli* strains **(A)**, and *cpa* in 3 *C. perfringens* strains **(B)**, and the data are presented as fold change of expression levels in nCFSM relative to non-fermented milk. The expression data of virulence genes for *E. coli* were normalized to *gapA* and *nusG, and C. perfringens* were normalized to *16sRNA* and *GAPDH*. Three independent replicates including two technical replicates each were performed. The data shown represents the mean and the error bars represent standard deviations. The stars indicate statistical significance at different levels: *P ≤ 0.05, ** *P* ≤ 0.01.

None of the nCFSM made from fermentations with each of the 61 individual LAB strains served to affect virulence gene expression in all three pathogens equally well. We therefore tested the possibility to obtain a nCFSM affecting the virulence genes in all the *E. coli* F5, *S*. Dublin and *C. perfringens* strains based on fermentations with mixtures of LAB strains. As the ability of LAB strains to grow and degrade milk may be affected by the presence of competing strains, we could not assume that the outcome from mixtures would simply be the sum of the results obtained from the individual nCFSMs. Hence, two cocktails were tested: Cocktail 1 consisted of a mix of NU-TRISH^®^ By-Mild and *B. longum* BL-15955 and cocktail 2 of a mix of COMBO4 with *B. longum* BL-15955. After growth in nCFSM from cocktail 1, virulence genes expression was reduced in the three tested *E*. coli F5 strains. The effect was particularly notable for *fanC* with 4.21 to 7.67 fold downregulation and *estA* with 3.83 to 5.36 fold regulation. Cocktail 2 also significantly decreased expression of *fanC* with 2.20 to 5.33 fold, and *estA* with 1.89 to 3.32 fold (Figure 6A). As seen in Figure 6B, nCFSM from cocktail 1 also significantly downregulated *cpa* in the 3 *C. perfringens* strains with 4.17 to 12.63 fold, while no significantly downregulation was found for this gene after growth in cocktail 2 (1.74 to 3.10 fold).

**Figure 7:**
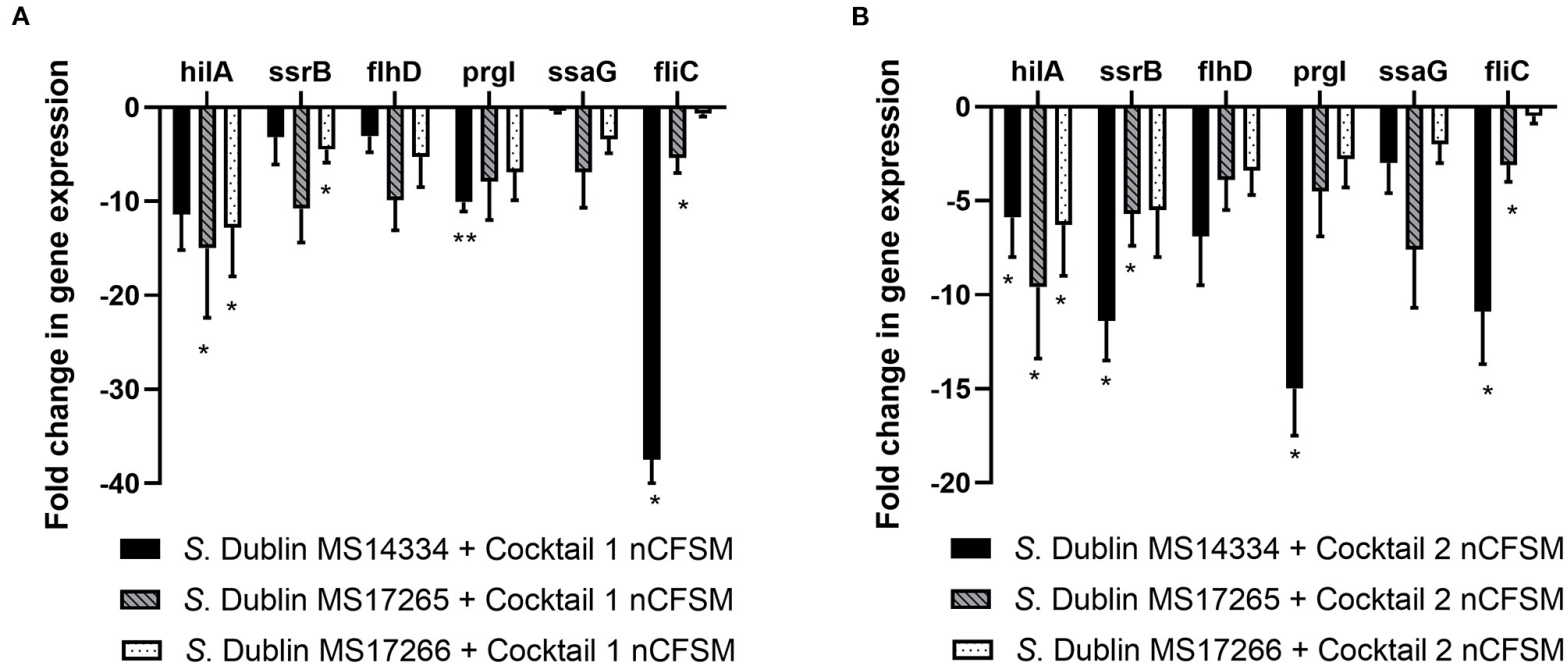
Effect of nCFSM from **(A)** cocktail 1 and **(B)** cocktail 2 on virulence gene expression in 3 *S. Dublin* strain. The expression data were normalized to validated reference gene *gapA* and presented as fold change of expression levels in nCFSM relative to non-fermented milk. Three independent replicates including two technical replicates each were performed. The data shown represents the mean and the error bars represent standard deviations. The stars indicate statistical significance at different levels: *P ≤ 0.05, ** *P* ≤ 0.01.

For *S*. Dublin, similar effects with significant downregulation of virulence genes were observed for the two cocktails. Cocktail 1 showed significant downregulation of *hilA* expression with 5.9 to 15.0 fold, *ssrB* expression with 3.2 to 10.8 fold, *flhD* with 3.1 to 9.9 fold, *prgI* with 7.9 to 15.0 fold, *ssaG* with 0.4 to 6.9 fold, and *fliC* with 0.7 to 37.5 fold in 3 different *S*. Dublin strains. The corresponding results for cocktail 2 was 5.9 to 9.6 fold (*hilA*), 5.5 to 11.4 fold (*ssrB*), 3.4 to 6.9 fold (*flhD*), 2.8 to 15.0 fold (*prgL*), 2.0 to 7.6 fold (*ssaG*), and 0.5 to 10.9 fold (*fliC*) (Figure 7).

## Discussion

It has previously been shown that certain LAB affects expression of virulence genes in enteropathogenic bacteria (Brovko et al., 2003; Medellin-Pena et al., 2007; Vinderola et al., 2007), and the use of this ability of LAB has been suggested as an effective measure to control entero-pathogens (Fooks and Gibson, 2002; Gusils et al., 2002). Based on this, the current study was concerned with the possible use of fermentation products from LAB strains to prevent expression of virulence genes necessary for induction of diarrhea in calves. This disease is caused by a multitude of pathogens, and focus was on infections caused by *Salmonella, E. coli* and *C. perfringens*, since they are the drivers of use of antimicrobials for this disease. Because anti-virulence approaches give rise to a milder evolutionary pressure and does not affect the ability of the pathogen to grow, it is less likely that resistance will be developed toward substances that affects virulence genes than toward conventional antibiotics (Rasko and Sperandio, 2010).

LAB are used in food processing because of their characteristic flavor formation and ability to lower the pH and to produce antimicrobial substances (Verluyten et al., 2003). The effect with certain LAB is seen even when using pH-neutral, cell-free supernatants of the strains (Bayoumi and Griffiths, 2010), suggesting that the effect on expression of virulence genes associated to substances produced by the LAB strains during growth, either products exported from the bacteria or degradation products of the growth medium. In the current study, we investigated the effect of pH neutralized, spent cow milk from 61 food grade starter cultures on virulence gene expression in the three bacteria associated with calf diarrhea. pH neutralization was done because there were measurable differences in the final pH after growth of LABs, and we wanted to rule out this factor as a contributor to the virulence inhibition. Milk is readily accessible in dairy farms where calf diarrhea is a disease problem, and fermented milk is already used to feed calves (Maldonado et al., 2018). In addition to the nutritional and specific antibacterial effect, milk components are known to have a variety of health benefits in humans, including opioid, antimutagenic, antimicrobial, and immunomodulating activity, in addition to their basic nutritional properties (Smacchi and Gobbetti, 2000). It has been reported that the consumption of milk fermented by lactic acid bacteria produces a specific humoral immune response, and may have effects on the digestive, cardiovascular and nervous systems (Matar et al., 2001; Korhonen, 2009). Whether similar effects can be seen in calves remains to be shown, but could be an additional benefit of the approach.

Previous studies have showed that metabolites produced by *L. helveticus* LH-2 and *L. acidophilus* LA-5 affected expression of *hilA, hilD, ssrA*, and *ssrB* in *Salmonella*, and *tir, ler, eaeA, fliC*, and *hlyB* in *E. coli* O157:H7 after growth in chemically defined media and milk (Medellin-Pena and Griffiths, 2009; Tellez et al., 2010; Bayoumi and Griffiths, 2012; Zeinhom et al., 2012). Here, we investigated the effects of neutralized cell-free spent milk (nCFSM) on a wider range of virulence genes in *Salmonella* and *E. coli*, and we included the main virulence gene shared between all types of *C. perfringens*. The most commonly observed fimbriae on ETEC from calves with diarrhea are F5 and F41 (Nagy and Fekete, 1999), and most bovine ETEC produce STa (Guth, 2000). In *Salmonella*, the gene *hilA* directly controls and activates all the genes of SPI1 for invasion (Lostroh et al., 2000), and *ssrB* is the main regulator of SPI2, which is responsible for systemic infection and replication inside macrophages and epithelial cells (Feng et al., 2004). Meanwhile, flagella are also involved in adhesion and host invasion, which is controlled by the regulator *flhD/C* (Kirov, 2003). Besides these three key regulator genes, three structural genes (*prgL, ssaG* and *fliC*) of each apparatus were also included in this study. Among *C. perfringens*, type A is the most common of all the *C. perfringens* types. The alpha-toxin encoded by *cpa* gene is the main lethal toxin of type A, and it is also produced by all other genotypes of *C. perfringens*. Type B and C which produce beta-toxin have been frequently reported in conjunction with calf diarrhea (Rings, 2004), but those types infection are not very common in calves. Though all types have the ability to produce the alpha-toxin, type A usually produces a greater amount (Katayama et al., 1993). In young calves, severe abomasitis associated with *C. perfringens* type A is characterized by sudden onset of disease, abomasal tympany, abdominal pain, and hemorrhagic diarrhea (Glenn Songer and Miskimins, 2005; Schlegel et al., 2012). Success in suppression these key virulence factors would putatively enable use of feeding with fermented milk as a way of preventing calf diarrhea caused by *S*. Dublin, ETEC F5, and *C. perfringens*. This would be a sustainable prevention measure, especially with the emerging problem of antibiotic-resistant strains.

In previous experiments, nCFSMs obtained from reconstituted milk powder have shown to be capable of modulating macrophage activity, as well as inhibit virulence genes expression (Brovko et al., 2003; Ding et al., 2005; Vinderola et al., 2007; Tellez et al., 2010, 2011), suggesting that reconstituted milk could be used as an alternative to fresh milk. In the current study, we compared the anti-virulence activity of nCFSMs made based on whole and reconstituted milk, and interestingly nCFSMs from whole milk showed better inhibition of virulence gene expression than reconstituted milk. The reason for this remains unknown. There was no observations to suggest that the LAB strains grew any different in the fresh compared to when they grew in re-constituted milk and the pathogens also grew equally well in nCFSM from the two sources (data not shown). It may be that some components of the milk, which are important for formation of the antibacterial principle is affected by the drying procedure used to make milk powder, as the chemical composition of reconstituted milk and fresh milk differ (Mehta, 2015). Further studies are needed to understand this difference.

Most previous studies on the effect of fermentation products from LAB on virulence gene expression have used 1% LAB inoculation level for fermentation (Brovko et al., 2003; Casey et al., 2007; Medellin-Pena et al., 2007; Medellin-Pena and Griffiths, 2009; Bayoumi and Griffiths, 2012), 2% (Matar et al., 2001; Vinderola et al., 2007), 4% (Ding et al., 2005), while 10% inoculation is less frequent (Tellez et al., 2010). We found that 10% inoculation level lead to nCFSMs with better anti-virulence activity. This may be because 10% inoculation resulted in more effective fermentation and thus lower pH and probably more production of the anti-virulence substances.

Different methodological strategies were used to screen for the ability of nCFSM to repress the virulence gene. A broad screen was carried out using bioluminescence activity of a plasmid borne promoter-reporter fusions *hilA::luxCDABE*. Bioluminescent reporter genes are considered a rapid tool for initial monitoring of gene expression; however, bioluminescence may be influenced by several factors, including other components of the medium. Previous observations using bioluminescent constructs suggest the occurrence of false positives and negatives with respect to virulence expression (Medellin-Pena et al., 2007; Delcenserie et al., 2012; Zeinhom et al., 2012; Guri et al., 2016). Therefore, in the current study, the samples were further analyzed using RT-qPCR to confirm the results and to have the opportunity to analyze the virulence expression of more genes in several pathogens. In this way it was shown that nCFSM from *B. infantis* BI-33361, MIX-2, and COMBO3 did not result in significant downregulation of *hilA* when measured by qPCR opposed to bioluminescent assay. Two *L. paracasei* strains, BGP1 and BGP2, together with *P. pentosaceus* PP-670 did not sustain growth of *Salmonella*. For this reason, these strains showed the highest inhibition of *hilA* expression in the bioluminescent assay, underlining the importance of controlling for growth, when analyzing effect of virulence gene expression. *hilA* expression varies with growth phase and is maximally expressed in late logarithmic phase (Miao et al., 1999; Mouslim and Hughes, 2014), thus comparison of effect of a substance on expression of *hilA* can only be done if the strains are in the same growth phase.

The 61 nCFSMs were made using LAB strains belonging to 17 genera, enabling analysis of whether the ability to affect virulence gene expression was a trait of strains from a particular genus. From the initial screening of the 61 strains, it seems that nCFSM of the *Enterococcus* genus had no effects on virulence gene expression. A previous study reported that *Enterococcus faecalis* Symbioflor^®^ downregulated virulence genes of *E. coli* O157:H7 through direct bacteria-bacteria interactions in a *Caenorhabditis elegans* model (Neuhaus et al., 2017), however, in line with our results, nCFSM with anti-virulence activity from *Enterococcus* genus has not been reported. Only three strains of *Enterococcus* were included in the current study, and care should be taken not to over-conclude. The observation may help in future to identify the active substances produced during fermentation, by comparing nCFSM from *Enterococcus* to nCFSM from the other genera.

A previous study showed that fractions extracted from *L. acidophilus* LA-5 cell-free spent medium were able to downregulate several virulence genes in verotoxigenic *E. coli* O157:H7 and that this could be used to decrease the severity of diarrhea in mice (Zeinhom et al., 2012). In the current study, virulence factors of enterotoxigenic *E. coli* (ETEC), namely *fanC* and *fim41a* genes encoding major subunits of the F5 and F41 fimbriae, and *estA* encoding STa enterotoxins, were investigated. nCFSM from the mixed culture NU-TRISH^®^ By-Mild and from *B. infantis* BI-33361, *B. longum* BL-15955, *L. buchneri* LB-881, *L. delbrueckii* subsp. *bulgaricus* LB20074, *L. reuteri* LR-33016, *P. acidilactici* PA-839 and *P. pentosaceus* PP-674, and *Pr. freudenreichii* PF-507 showed anti-virulence activity when only one *E. coli* strain was assayed, however, when extra strains were included, only nCFSM from *B. longum* BL-15955 and *L. reuteri* LR-33016 stably reduced the expression of the three genes in all tested *E. coli* strains. These LAB strains were thus indicated as the best candidates to control calf diarrhea caused by ETEC F5 and F41. *L. johnsonii* F19785 has been reported to reduce the colonization and shedding of *C. perfringens* in poultry as a defined competitive exclusion agent (La Ragione et al., 2004). In the current study, nCFSM from *B. longum* BL-15955, *L. buchneri* LB-881, *L. johnsonii* LJ-10533, *L. rhamnosus* LR-20021, *L. lactis* LL-995, and the multi-strain cultures COMBO3 and COMBO4 showed potent downregulation of *cpa* expression in one *C. perfringens* strain. Due to an inhibitory effect on virulence genes in *E. coli, B. longum* BL-15955, *L. buchneri* LB-881 and COMBO4 were selected for testing with more *C. perfringens* strains. Only *B. longum* BL-15955 showed significant downregulatory effect on the *cpa* gene of all tested *C. perfringens* strains.

With respect to *Salmonella*, previous studies have included effect on *hilA* and *ssrB* encoding key regulators of TTSSs on SPI-1 and SPI-2, and for these virulence factors, there is evidence that the downregulation affects virulence in cell cultures and mice. For example, nCFSM from *B. bifidum* ATCC 29521 and *L. helveticus* LH-2 affected attachment and invasion of *Salmonella in vitro* and *in vivo* through inhibition of these virulence genes (Tellez et al., 2011; Bayoumi and Griffiths, 2012). In the current study, we revisited these key regulator genes, and in addition we included the main regulator of the flagella operon, *flhD*. For each of these important virulence associated systems, we further included a structural genes (*prgI* of T3SS-1, *ssaG* of T3SS-2 and *fliC* of the flagella) to have a stronger argument for a possible effect. Previous studies have been concerned with *S*. Typhimurium, while we have focused on *S*. Dublin, the main serovar involved in calf diarrhea. Many LAB strains showed inhibition of these genes, suggesting that the factors which affect virulence gene expression in *Salmonella* are relatively commonly formed during lactic acid bacteria fermentation of milk. Characteristically, when downregulation was seen in regulator genes, this was always followed by at least the same trend in the corresponding structural gene (Supplementary Dataset S1). We were primarily concerned with finding LAB strains which inhibit a broad selection of virulence genes and in as many strains as possible. nCFSM of two multi-strain cultures, NU-TRISH^®^ By-Mild and COMBO4, and the single strain culture, *L. helveticus* CNRZ32, showed significant downregulation in all tested *Salmonella* strains.

In a previous study, administration of *L. acidophilus* alone showed no beneficial effects on lambs infected with *E. coli* O157:H7, while feeding the lambs with a mixture of *L. acidophilus* and *E. faecium* significantly lowered numbers of this pathogenic strain (Lema et al., 2001). The greatest reduction in numbers was seen with the use of a mixture of *L. acidophilus, E. faecium, L. casei, L. fermentum*, and *L. plantarum* (Lema et al., 2001). A five-strain probiotic combination consisted of two *L. murinus* strains and one strain of each *L. salivarius, L. pentosus* and *P. pentosaceous* also reduced pathogen shedding and alleviated diarrhea in pigs challenged with *S*. Typhimurium (Casey et al., 2007). In line with the beneficial effects of multi-strains probiotics, nCFSM made from multi-strains cultures generally showed good effect on virulence gene expression. Thus, NU-TRISH^®^ By-Mild consisting of *B. lactis* BL-15954, *L. delbrueckii* subsp. *bulgaricus* and *S. thermophilus*, and COMBO4 consisting of *L. delbrueckii* subsp. *bulgaricus* and *S. thermophilus* both showed significant anti-virulence activity. The basis of the multifactorial mode of action of multi-strain probiotics remains largely un-clarified, but it may be that modulation of virulence is an important feature in their protection against enteric infections.

The experience from probiotics and the results of our initial screening indicated the possibility of using a multi-strain combination to control more than one pathogenic species. In order to identify the best candidate to reduce pathogens associated with calf diarrhea, cocktails were tested. A cocktail with NU-TRISH^®^ By-Mild, which had excellent effect on genes in *S. Dublin* and *B. longum* BL-15955 with good effect on ETEC F5 and *C. perfringens* was shown to be very promising, and should be taken into further testing. This four-strain combination showed significant downregulation of virulence genes in all tested pathogens. However, the level of reduction was reduced for *S. Dublin* compared with NU-TRISH^®^ By-Mild alone, underlying that combination of strains do not just have the added effects of each strain individually.

The observation that fermented milk with this combination down regulates virulence genes in the major bacterial pathogens associated with calf diarrheae opens the possibility that feeding with this milk may prevent the pathogens from causing disease in the calfes. However, there is a long way from showing this effect in the laboratory to concluding on the effect on field conditions. Not only will there be variation in intake of the fermented milk, but their effect on virulence genes may also be modified once they reach the active sites in the intestine. Further studies should thus be undertaken to investigate whether the principle will work *in vivo* by feeding calves with cow milk fermented with this four-strain combination.

## Conclusions

In summary, the data presented in this study confirms that milk fermented by selected LAB strains may affect virulence genes in bacteria. The study performed a broad screening of single strains as well as multi-strains of LAB strains. In addition to previous studies, it included ETEC and *C. perfringens* virulence genes, and it focused on inhibition of virulence genes in bacteria relevant to induction of diarrhea in calves. A four-strain combination with *B. lactis* BL-15954, *L. delbrueckii* subsp. *bulgaricus, S. thermophilus* and *B. longum* BL-15955 was identified which gave stable anti-virulence effect in *S*. Dublin, ETEC F5 and *C. perfringens*, and these observations warrant further *in vivo* validation. Clearly, additional research must be conducted to demonstrate that this can lead to reduced incidence of diarrhea in calves and identify molecules responsible for the observed activity and determine the mechanisms whereby nCFSM inhibits the activity of the virulence genes.

## Data Availability Statement

The datasets presented in this study can be found in online repositories. The names of the repository/repositories and accession number(s) can be found below: European Nucleotide Archive - PRJEB46410.

## Author Contributions

GL, JO, MK, SA, and AJ have participated in the design of the study. GL and MK carried out the experiments. GL drafted the manuscript. All authors commented on and approved the final manuscript.

## Funding

This work was funded by the GUDP Development and Demonstration Program through Grant No. 1167960.

## Conflict of Interest

The authors declare that the research was conducted in the absence of any commercial or financial relationships that could be construed as a potential conflict of interest.

## Publisher's Note

All claims expressed in this article are solely those of the authors and do not necessarily represent those of their affiliated organizations, or those of the publisher, the editors and the reviewers. Any product that may be evaluated in this article, or claim that may be made by its manufacturer, is not guaranteed or endorsed by the publisher.
