# Supplementary material for: Inhibition of Virulence Gene Expression in Salmonella Dublin, Escherichia coli F5 and Clostridium perfringens Associated With Neonatal Calf Diarrhea by Factors Produced by Lactic Acid Bacteria During Fermentation of Cow Milk
Source: Front Microbiol. 2022 May 12;13:828013. doi: 10.3389/fmicb.2022.828013 (PMC9134014; doi:10.3389/fmicb.2022.828013)
Supplement: Supplementary file 1 [file Data_Sheet_1.zip › Supplementary Datasheet 1.docx]

Establish culture collection

(61 LAB)

Optimal fermentation protocol

Preparation of nCFSM

10 best candidate

nCFSMs

Test anti-virulence activity in 3 *S*. Dublin, 4 *E. coli* F5, and

4 *C. perfringens* by RT-qPCR to verify strain coverage

Bioluminescence assay

RT-qPCR

Preparation of nCFSM cocktails

Test anti-virulence activity in 3 *S*. Dublin, 3 *E. coli* F5, and 3 *C. perfringens* to verify strain coverage

*E. Coli* E21-79

*S*. Dublin JEO3665

*C. perfringens* C4-5

*S*. Typhimurium LT2 *hilA*::*luxCDABE*

Potential candidates for prevention of neonatal calf diarrhea caused by *S.* Dublin, *E. coli* F5, and *C. perfringens*

Initial screening

RT-qPCR

Bioluminescence assay

*S*. Dublin

*S*. Typhimurium LT2 *hilA*::*luxCDABE*

Best nCFSM with stable anti-virulence effect

**Supplementary Figure S1**. Schematic overview of general experimental design.

**
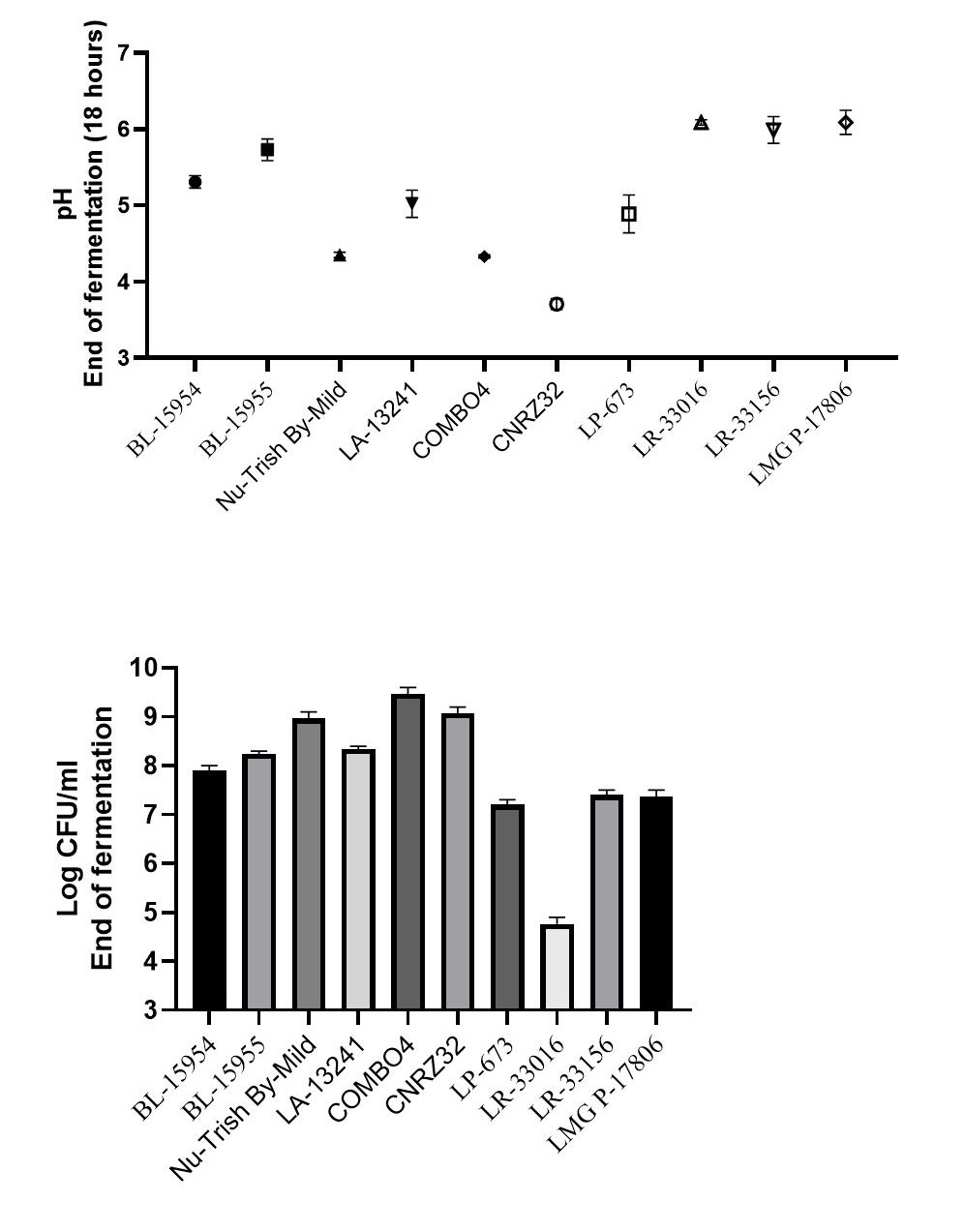
**

**Supplementary Figure S2**. Top panel shows Log CFU after 18 hours of growth in milk and Bottoom panel shows the resulting pH in the milk.

**Supplementary Table S1**. Primers used in this study.

| Genes | Functions | Sequence (5’-3’) | Efficiency |
| --- | --- | --- | --- |
| *fanC* | Major F5 fimbrial subunit | F: CACTACGGCTGAATACACTC  R: TGACTAAGAAGGATGCTGAAG | 1.96 |
| *F41* | Major F41 fimbrial subunit | F: TAGCAGCGAAGATGAGTGA  R: AATGACAAAGGAACAGAAAGAGT | 1.99 |
| *estA* | Heat-stable enterotoxin | F: CAACTGAATCACTTGACTCTTC  R: GCACAGGCAGGATTACAA | 2.08 |
| *hilA* | Transcriptional regulator of SPI-1 | F: CATACATTGGCGATACTTCCT  R: GCGGCAGTTCTTCGTAAT | 2.05 |
| *prgI* | Needle subunit regulated by hilA | F: AATGGCAGCATCAATATCCT  R: CGGCGTATCAGAGTAAGC | 2.03 |
| *ssrB* | Transcriptional regulator of SPI2 | F: AAGCAGTAGTCAGCAAGTTC  R: AAGTAAGCAGTTGATGATTGGT | 1.98 |
| *ssaG* | A gene that encoded the T3SS in SPI2 | F: GATTCCACTAAGCATATCCTTGA  R: GCCTTACAGCAGTATTCTACAT | 2.04 |
| *flhD* | Transcriptional regulator of flagella genes | F: CCGTTTGACTCAGGATTCG  R: CCGTATCGTCCACTTCATTG | 1.99 |
| *fliC* | Flagella filament protein | F: AAGGTGGTAAGGAAGGAGAT  R: TTGATTGTAAGGTAGCAGCATTA | 1.98 |
| *cpa* | Alpha toxin | F: TAGTTGGGATGATTGGGATTATG  R: CCTCTGATACATCGTGTAAGAAT | 2.03 |
| *gapA* | Glyceraldehyde-3-phosphate dehydrogenase, used as housekeeping gene | F: ACTGACTGGTATGGCGTTCC  R: GTTGCAGCTTTTTCCAGACG | 2.07 |
| *nusG* | Transcription termination / antitermination protein, used as housekeeping gene | F: CAGCAGGTTGGCGATAAG  R: CACTTTCAGGCGAGACTTC | 2.36 |
| GAPDH | Glyceraldehyde-3-phosphate dehydrogenase, used as housekeeping gene | F: CAAGGAGTAAGAGCCCCTGG  R: GGTACATGACGAGGCAGGTC | 2.27 |
| *16S rRNA* | 16S ribosomal RNA, used as housekeeping gene | F: CGCATAACGTTGAAAGATGG  R: TGGCACGTAGTTAGCCGT | 2.00 |

**Supplementary Table S2**. Effect of nCFSM on virulence gene expression in *S*. Dublin JEO3665, *E. coli* E21-79 and *C. perfringens* C4-5. The data is based on one biological replicate of nCFSM with technical replicates.

| **Strains ^a^** | **Product Code ^b^** | ***hilA* lux ^c^** | ***S*. Dublin**  **JEO3665 ^d^** | | | | ***E. coli***  **E21-79 ^d^** | | | ***C. perfringens***  **C4-5 ^d^** |
| --- | --- | --- | --- | --- | --- | --- | --- | --- | --- | --- |
|  |  |  | *hilA* | *ssrB* | *fliC* | *prgI* | *fanC* | *fim41a* | *estA* | *Cpa* |
| (+) *Bifidobacterium lactis*,  *Lactobacillus delbrueckii* subsp*. bulgaricus*,  *Streptococcus thermophilus* | NU-TRISH^®^  By-Mild | -2.1 | -8.1 | -51.5 | -279.2 | -614.5 | -3.0 | -2.2 | -4.3 | -0.6 |
| *Bifidobacterium animalis* subsp*. lactis* | BLC1 | -2.1 | -7.8 | -2.5 | -3.8 | -0.4 | -1.0 | -0.3 | -0.3 | -0.8 |
| *Bifidobacterium animalis* subsp*. lactis* | BL-15954 | -1.7 | -2.1 | -7.0 | -45.2 | -174.7 | -0.9 | -0.5 | -0.4 | -0.2 |
| *Bifidobacterium longum* subsp*. infantis* | BI-33361 | -5.9 | -1.7 | -2.9 | -0.7 | -0.6 | -13.7 | -4.5 | -5.0 | -0.4 |
| *Bifidobacterium longum* subsp*. longum* | BL-15955 | -2.3 | -1.3 | -6.3 | -139.4 | -158.3 | -5.8 | -6.4 | -3.0 | -3.6 |
| *Enterococcus faecium-*669 | EF-669 | -1.3 | -2.1 | -1.1 | -2.8 | -0.9 | -0.8 | -1.2 | -0.6 | -0.7 |
| *Enterococcus faecium-*202 | EF-202 | -1.8 | -2.5 | -2.2 | -0.3 | -0.3 | -1.2 | -0.9 | -0.3 | -1.1 |
| *Enterococcus faecium-*339 | EF-339 | -1.3 | -1.4 | -2.7 | -0.4 | -0.1 | -3.4 | -3.7 | -1.7 | -1.3 |
| *Kocuria varians* | KV | -1.3 | -0.9 | -1.7 | -36.1 | -33.2 | -2.3 | -3.0 | -1.1 | -3.2 |
| (+) *Kocuria varians*,  *Latilactobacillus curvatus,*  *Staphylococcus carnosus* | RD-1 | -1.0 | -2.7 | -6.7 | -31.7 | -73.8 | -0.9 | -3.9 | -7.1 | -4.3 |
| *Lactobacillus acidophilus* | LA-3 | -2.6 | -4.5 | -8.9 | -5.4 | -5.5 | -2.3 | -1.5 | -1.9 | -1.1 |
| *Lactobacillus acidophilus,* | LA-13241 | -2.0 | -2.5 | -4.6 | -1.2 | -0.3 | -3.0 | -1.7 | -1.8 | -0.3 |
| *Lactobacillus acidophilus* | LA-20079 | -1.7 | -1.7 | -4.4 | -3.3 | -0.3 | -1.2 | -1.5 | -1.2 | -1.9 |
| *Ligilactobacillus animalis-*506 | LA-506 | -1.7 | -2.2 | -3.1 | -2.1 | -0.4 | -2.4 | -1.4 | -0.9 | -2.4 |
| *Lentilactobacillus buchneri-*881 | LB-881 | -2.4 | -1.7 | -2.9 | -1.3 | -1.1 | -12.7 | -3.1 | -4.8 | -3.1 |
| *Lactobacillus delbrueckii* subsp*. Bulgaricus* | SP5 | -2.1 | -1.8 | -5.2 | -7.4 | -3.1 | -0.7 | -0.4 | -0.5 | -0.6 |
| (+) *Lactobacillus delbrueckii* subsp*. bulgaricus*,  *Streptococcus thermophilus* | COMBO4 | -2.0 | -6.8 | -30.8 | -65.3 | -159.0 | -1.9 | -1.3 | -0.7 | -3.8 |
| *Levilactobacillus brevis* | LB | -1.9 | -2.5 | -4.9 | -3.2 | -0.2 | -1.6 | -0.5 | -3.8 | -0.8 |
| *Lacticaseibacillus paracasei* subsp*. paracasai,* | LP-33451 | -1.5 | -2.5 | -4.6 | -0.4 | -0.3 | -1.3 | -0.8 | -0.4 | -1.0 |
| *Lacticaseibacillus casei* | BGP93 | -1.7 | -2.6 | -11.3 | -49.7 | -6.1 | -2.4 | -2.3 | -0.9 | -1.7 |
| *Latilactobacillus curvatus* | LC | -1.3 | -0.9 | -1.7 | -36.1 | -33.2 | -2.3 | -3.0 | -1.1 | -3.2 |
| *Lactobacillus delbrueckii* | LB-20074 | -1.8 | -12.9 | -2.8 | -33.6 | -4.8 | -65.6 | -24.6 | -63.9 | -2.2 |
| (+) *Lactobacillus delbrueckii* subsp*. bulgaricus*,  *Streptococcus thermophilus* | COMBO1 | -4.7 | -5.5 | -12.3 | -198.8 | -174.0 | -2.5 | -2.0 | -1.6 | -0.2 |
| (+) *Lactobacillus delbrueckii* subsp. *bulgaricus*,  *Streptococcus thermophilus* | COMBO2 | -2.0 | -14.1 | -2.7 | -3.7 | -4.0 | -1.2 | -0.6 | -0.3 | -1.6 |
| *Limosilactobacillus fermentum* | LF | -1.5 | -1.6 | -3.1 | -0.3 | -0.1 | -1.9 | -1.4 | -0.8 | -0.9 |
| *Lactobacillus helveticus* | LH521 | -2.8 | -5.3 | -9.3 | -1.8 | -0.9 | -1.7 | -1.7 | -1.0 | -0.9 |
| *Lactobacillus helveticus* | CNRZ32 | -16.2 | -10.9 | -20.4 | -10.7 | -2.6 | -2.1 | -3.4 | -2.0 | -0.6 |
| *Lactobacillus helveticus-*02 | LH-02 | -2.4 | -2.5 | -3.9 | -7.0 | -17.2 | -0.9 | -0.6 | -0.5 | -0.4 |
| *Lactobacillus* *johnsonii* | LJ-10533 | -1.2 | -2.4 | -6.9 | -162.1 | -299.2 | -1.1 | -2.5 | -1.6 | -3.2 |
| *Lacticaseibacillus paracasei* subsp*. paracasai* | BGP1 | -50.2 | -6.1 | -20.4 | -70.2 | -60.0 | -3.1 | -1.5 | -1.7 | -0.4 |
| *Lacticaseibacillus paracasei* subsp*. paracasai* | BGP2 | -50.2 | NG^e^ | NG^e^ | NG^e^ | NG^e^ | NG^e^ | NG^e^ | NG^e^ | -0.5 |
| *Lacticaseibacillus paracasei* subsp*. paracasai* | LMG P-17806 | -1.7 | -0.7 | -1.8 | -15.7 | -10.7 | -6.1 | -4.3 | -2.5 | -3.0 |
| *Lacticaseibacillus paracasei* subsp*. Paracasai* | LP-20006 | -1.6 | -1.8 | -0.9 | -17.9 | -9.0 | -2.3 | -1.5 | -1.4 | -3.0 |
| *Lactiplantibacillus plantarum* | LPAL | -2.3 | -2.6 | -4.0 | -4.1 | -2.1 | -1.6 | -0.4 | -0.5 | -0.2 |
| *Lactiplantibacillus plantarum­-*672 | LP-672 | -1.4 | -4.7 | -0.9 | -0.9 | -0.7 | -1.5 | -0.6 | -0.7 | -1.5 |
| *Lactiplantibacillus plantarumi-*673 | LP-673 | -1.4 | -2.2 | -5.0 | -155.4 | -257.5 | -2.2 | -2.9 | -1.6 | -1.1 |
| *Lactiplantibacillus plantarum-*072 | LP-072 | -1.3 | -1.1 | -3.9 | -8.0 | -0.7 | -4.8 | -2.4 | -1.9 | -0.8 |
| *Lactiplantibacillus plantarum* | LP-20174 | -1.1 | -2.7 | -2.1 | -1.2 | -3.4 | -2.1 | -7.3 | -2.3 | -2.2 |
| *Limosilactobacillus reuteri* | LR-33016 | -4.5 | -5.8 | -40.5 | -275.5 | -250.0 | -16.6 | -6.7 | -19.6 | -1.4 |
| *Limosilactobacillus reuteri* | LR-20016 | -1.2 | -1.9 | -9.5 | -223.7 | -355.4 | -2.3 | -2.9 | -5.0 | -1.9 |
| *Lacticaseibacillus rhamnosus* | IMC 501 | -1.7 | -3.2 | -0.9 | -0.5 | -0.2 | -2.8 | -1.1 | -1.8 | -0.5 |
| *Lacticaseibacillus rhamnosus* | LR-33156 | -1.1 | -0.7 | -1.4 | -154.7 | -43.4 | -2.5 | -2.8 | -1.9 | -2.3 |
| *Lacticaseibacillus rhamnosus* | LR-20021 | -1.8 | -5.0 | -1.8 | -24.7 | -1.6 | -7.2 | -1.6 | -2.5 | -6.0 |
| *Lacticaseibacillus rhamnosus* | SP1 | -1.6 | -3.0 | -4.4 | -6.1 | -2.5 | -2.0 | -4.3 | -3.9 | -0.8 |
| *Lactococcus lactis*-955 | LL-995 | -2.7 | -2.5 | -2.7 | -5.5 | -0.5 | -1.5 | -7.0 | -8.1 | -7.9 |
| *Lactococcus lactis*-671 | LL-671 | -1.2 | -1.5 | -1.4 | -3.9 | -147.5 | -1.4 | -9.5 | -9.7 | -1.9 |
| (+) *Lactococcus cremoris,*  *Leuconostoc* spp.,  *Lactococcus lactis* subsp*. lactis*,  *Lactococcus lactis* subsp. *lactis*  *biovar diacetylactis* | MIX-1 | -2.3 | -1.2 | -3.2 | -0.3 | -0.2 | -3.2 | -5.1 | -1.2 | -1.5 |
| (+) *Lactococcus cremoris,*  *Leuconostoc* spp.,  *Lactococcus lactis* subsp. *lactis*,  *Lactococcus lactis* subsp. *lactis*  *biovar diacetylactis* | MIX-2 | -2.9 | -0.9 | -2.8 | -3.0 | -0.3 | -2.0 | -1.3 | -0.3 | -2.4 |
| (+) *Lactococcus cremoris*,  *Lactococcus lactis* subsp. *lactis*,  *Lactococcus lactis* subsp. *lactis*  *biovar diacetylactis*,  *Leuconostoc spp.* | MIX-3 | -2.0 | -0.9 | -4.1 | -175.9 | -244.9 | -4.0 | -2.8 | -4.1 | -1.4 |
| *Lactococcus lactis* spp. *lactis* biovar diacetylactis (*L. diacetylactic*) | LD | -2.3 | -1.6 | -3.0 | -196.9 | -243.0 | -1.5 | -1.8 | -1.0 | -1.5 |
| (+) *Latilactobacillus sakei* subsp*. sakei*,  *Staphylococcus carnosus* | LD-20 | -1.4 | -29.4 | ND | -16.3 | -1.5 | -0.8 | -1.0 | -2.2 | -1.5 |
| (+) *Lactobacillus delbrueckii* subsp. *bulgaricus*,  *Streptococcus thermophilus* | COMBO3 | -6.3 | -1.6 | -3.3 | -4.2 | -0.9 | -4.8 | -1.8 | -2.0 | -5.3 |
| *Pediococcus acidilactici*-839 | PA-839 | -1.7 | -2.5 | -5.7 | -7.9 | -0.8 | -4.3 | -4.0 | -2.7 | -0.7 |
| *Pediococcus pentosaceus*-354 | PP-354 | -2.2 | -4.0 | -4.5 | -6.7 | -8.5 | -1.3 | -8.3 | -2.8 | -1.8 |
| *Pediococcus pentosaceus*-670 | PP-670 | -3.9 | NG^e^ | NG^e^ | NG^e^ | NG^e^ | NG^e^ | NG^e^ | NG^e^ | NG^e^ |
| *Pediococcus pentosaceus*-674 | PP-674 | -1.6 | -2.5 | -4.2 | -30.7 | -1.6 | -3.1 | -3.2 | -4.4 | -0.7 |
| *Propionibacterium freudenreichii* ssp. *Shermanii* | PF7 | -1.4 | -1.1 | -3.3 | -0.6 | -0.5 | -1.0 | -0.4 | -0.3 | -1.1 |
| *Propionibacterium freudenreichii* ssp. *Shermanii* | PF8 | -1.2 | -1.6 | -3.5 | -0.5 | -0.2 | -1.0 | -0.7 | -0.3 | -3.0 |
| *Propionibacterium freudenreichii* ssp. *Shermanii* | PB-1 | -2.0 | -5.2 | -5.1 | -1.6 | -0.2 | -0.3 | -0.7 | -0.3 | -0.7 |
| *Propionobacterium freudenreichii-*507 | PF-507 | -2.1 | -3.5 | -2.6 | -6.6 | -20.9 | -6.1 | -24.5 | -10.1 | NG |
| *Staphylococcus carnosus* | SC | -3.6 | -5.2 | -22.5 | -8.8 | -0.7 | -2.9 | -1.9 | -1.4 | -1.3 |

^a^ Multi-strain cultures indicated with (+)

^b^ The 10 nCFSMs inhibit better virulence genes expression chose to test on more pathogenic strains are shown in Bold.

^c^ Data are presented as fold change in nCFSM from 61 different LAB cultures induced bioluminescence of *S*. Typhimurium *hilA*::*lux*CDABE relative to bioluminescence levels with non-fermented milk control.

^d^ Effect of nCFSM on virulence gene expression in *S*. Dublin JEO3665, *E. coli* E21-79 and *C. perfringens* C4-5 by RT-qPCR.

e NG, not growth.
